# Pillararenes Trimer for Self-Assembly

**DOI:** 10.3390/nano10040651

**Published:** 2020-03-31

**Authors:** Huacheng Zhang, Zhaona Liu, Hui Fu

**Affiliations:** 1 School of Chemical Engineering and Technology, Xi’an Jiaotong University, Xi’an 710049, China; 2 Medical School, Xi’an Peihua University, Xi’an 710125, China; zhaonaliu@peihua.edu.cn; 3 College of Science, China University of Petroleum, Qingdao 266580, China; fuhui@upc.edu.cn

**Keywords:** pillararenes trimers, supramolecular interactions, self-assembly, synthesis, applications

## Abstract

Pillararenes trimer with particularly designed structural geometry and excellent capacity of recognizing guest molecules is a very efficient and attractive building block for the fabrication of advanced self-assembled materials. Pillararenes trimers could be prepared via both covalent and noncovalent bonds. The classic organic synthesis reactions such as click reaction, palladium-catalyzed coupling reaction, amidation, esterification, and aminolysis are employed to build covalent bonds and integrate three pieces of pillararenes subunits together into the “star-shaped” trimers and linear foldamers. Alternatively, pillararenes trimers could also be assembled in the form of host-guest inclusions and mechanically interlocked molecules via noncovalent interactions, and during those procedures, pillararenes units contribute the cavity for recognizing guest molecules and act as a “wheel” subunit, respectively. By fully utilizing the driving forces such as host-guest interactions, charge transfer, hydrophobic, hydrogen bonding, and C–H^…^π and π–π stacking interactions, pillararenes trimers-based supramolecular self-assemblies provide a possibility in the construction of multi-dimensional materials such as vesicular and tubular aggregates, layered networks, as well as frameworks. Interestingly, those assembled materials exhibit interesting external stimuli responsiveness to e.g., variable concentrations, changed pH values, different temperature, as well as the addition/removal of competition guests and ions. Thus, they could further be used for diverse applications such as detection, sorption, and separation of significant multi-analytes including metal cations, anions, and amino acids.

## 1. Introduction

Self-assembly has attracted much more attention in fundamental researches of material science and interesting applications to practical engineering areas [1,2], e.g., providing not only inspiring methodological strategy in processing, but also solid functional materials to balance morphologies and properties [3]. Building ideal self-assembly begins from the molecular level [2,4,5], i.e., designing the structural geometry of molecules, modifying them with proper functional moieties, introducing and choosing proper inter/intramolecular interactions, as well as controlling the behavior of assembled molecules. Thus, designing appropriate building blocks from the molecular level is very significant in the construction of multi-dimensional self-assembly [2].

**Chart 1:**
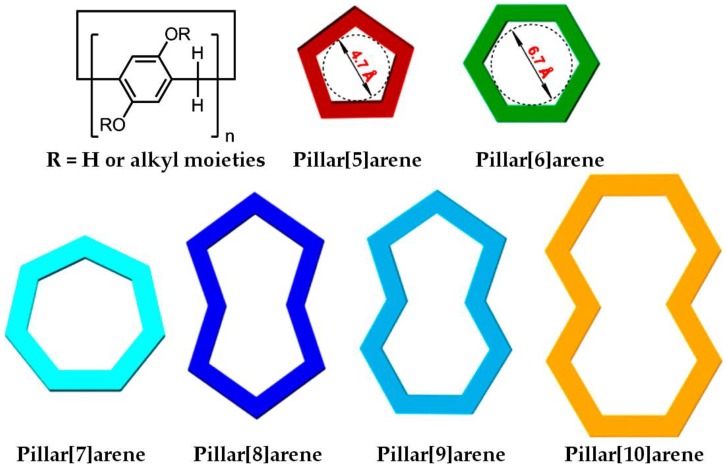
Structural and cartoon representations of typical pillar[n]arenes (*n* = 5—10). Reproduced with permission from [1]. Copyright American Chemical Society, 2015.

Macrocycles is a kind of particular cyclic oligomers with the hollow cavity for recognizing guest molecules [6,7], providing a possibility to introduce more functional and sensitive moieties for the fabrication of building blocks, as well as controllable self-assembly through supramolecular interactions such as host-guest interactions [1,8,9,10]. Pillararenes is a rising star in macrocycles (Chart 1) [11], due to its high synthesis yield and convenient modifications [12]. Different from other macrocycles, pillararenes composed by repeated phenol subunits possesses the electron-rich cavity and more rigid chemical structures, leading to its unique physiochemical properties such as planar chirality, as well as recognition towards neutral and electron-deficient guests [13,14]. Interestingly, functionalized pillararenes monomers and dimers have been used as the building blocks to construct self-assemblies such as vesicular and polymeric architectures with small guest molecules as the template [15,16,17,18], but their morphologies are still limited.

To enlarge the family of assemblies with controllable multi-dimensional morphologies such as two-dimensional materials, pillararenes trimers have been designed and have attracted much more attentions recently. Different from pillararenes monomers and dimers, pillararenes trimers integrate three pieces of pillararenes units together via a “core” bridge, contribute a planar geometry as building blocks for advanced self-assemblies, and generate unique physiochemical properties such as aggregation-induced absorption [19].

**Scheme 1:**
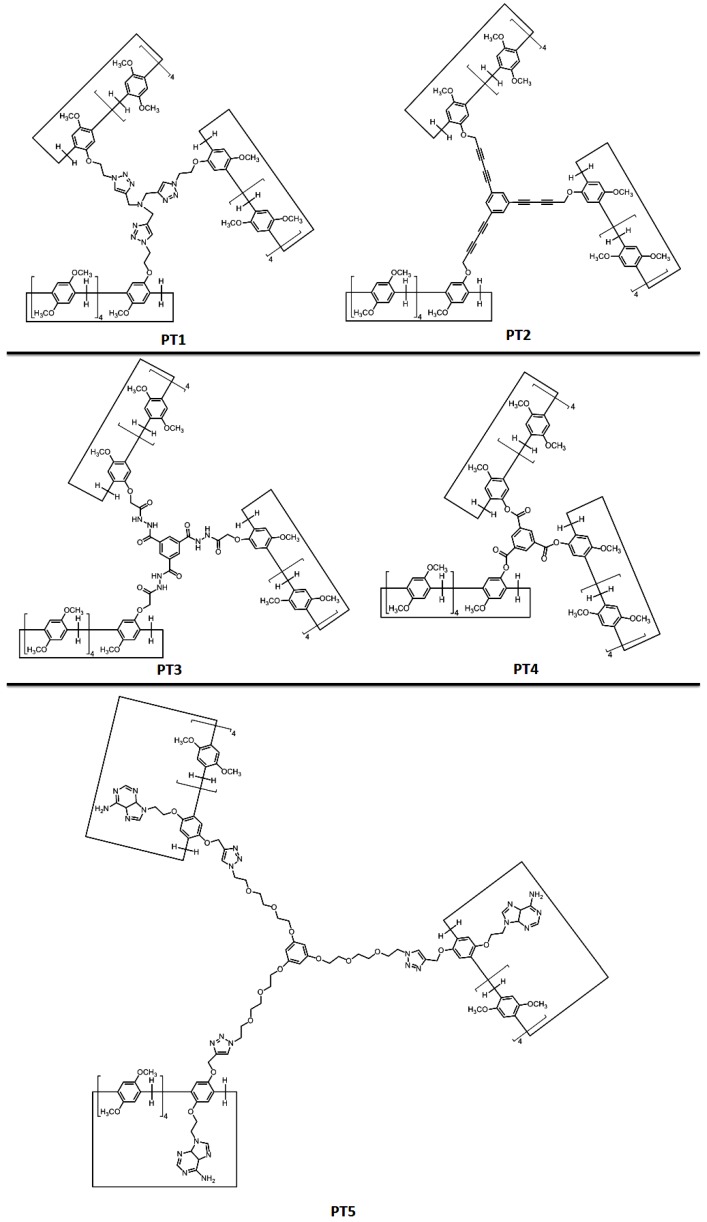

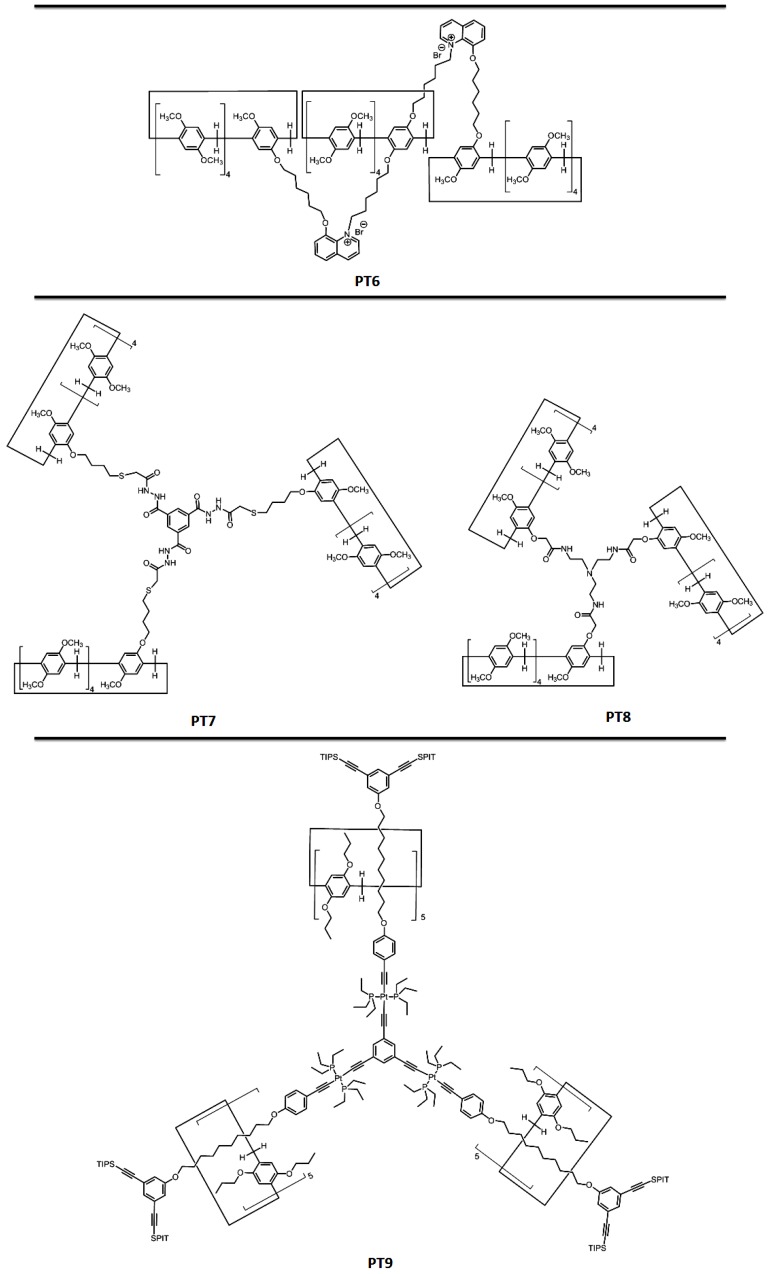

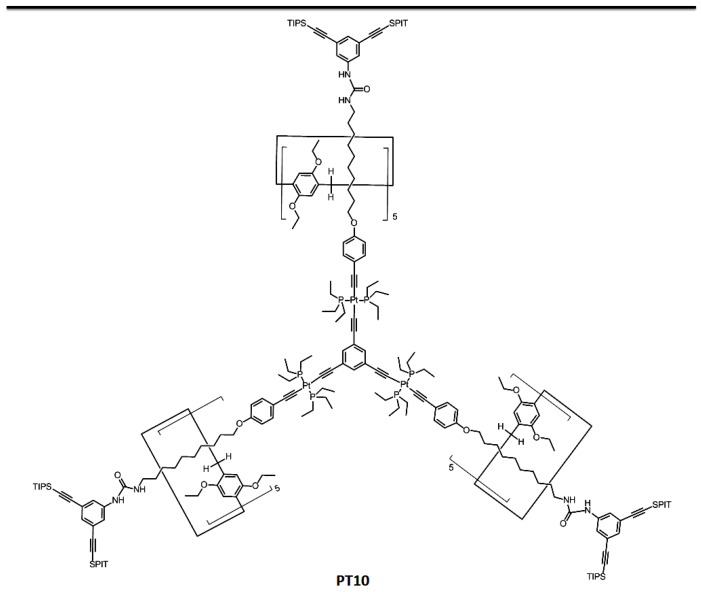
Chemical structures of pillararenes trimers (**PT1**–**PT10**).

**Scheme 2:**
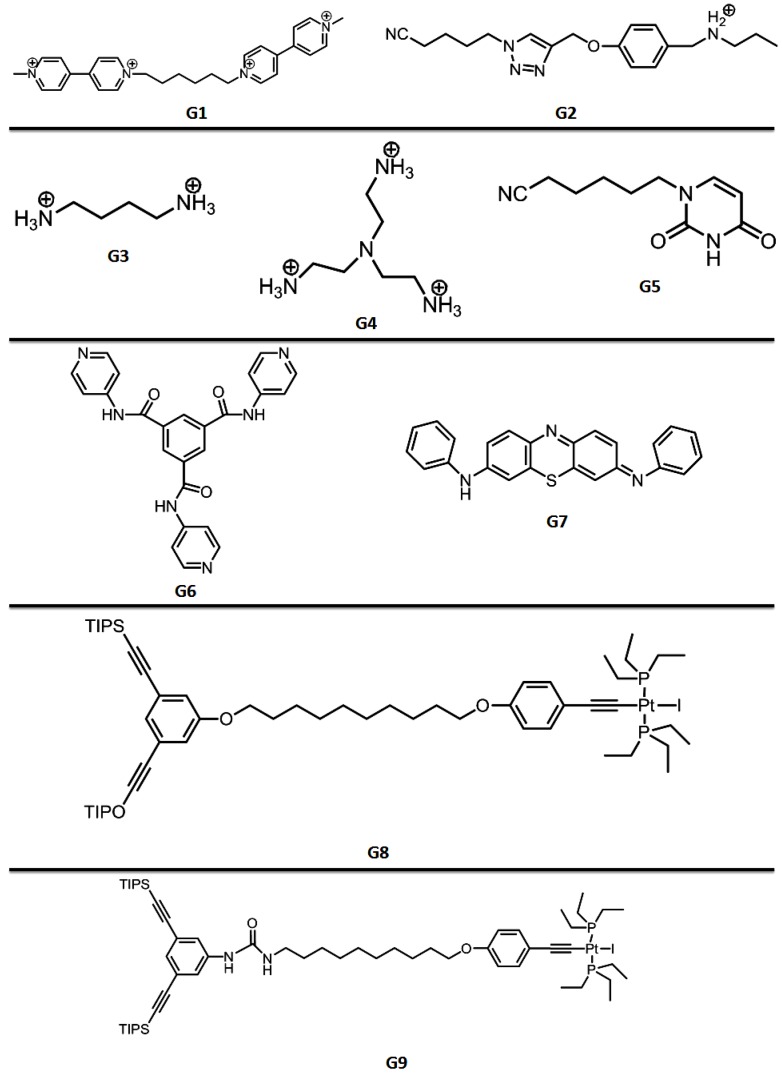
Chemical structures of guest molecules (**G1**–**G9**).

**Scheme 3:**
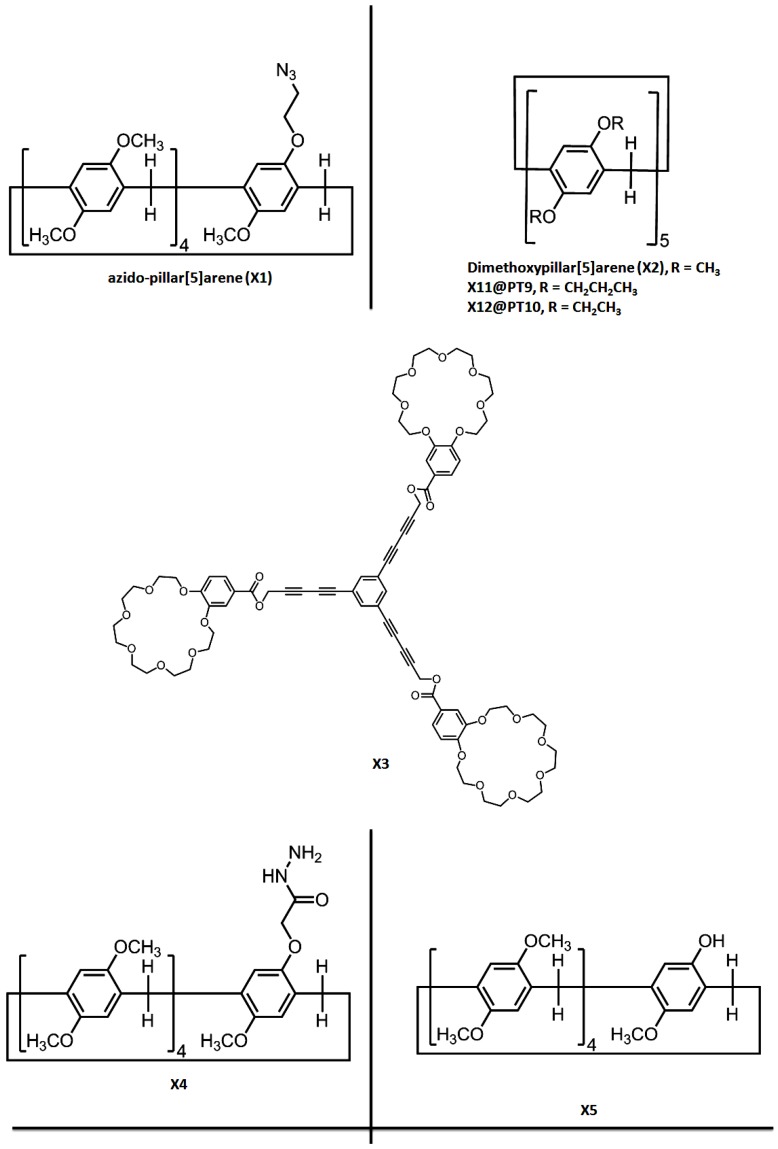

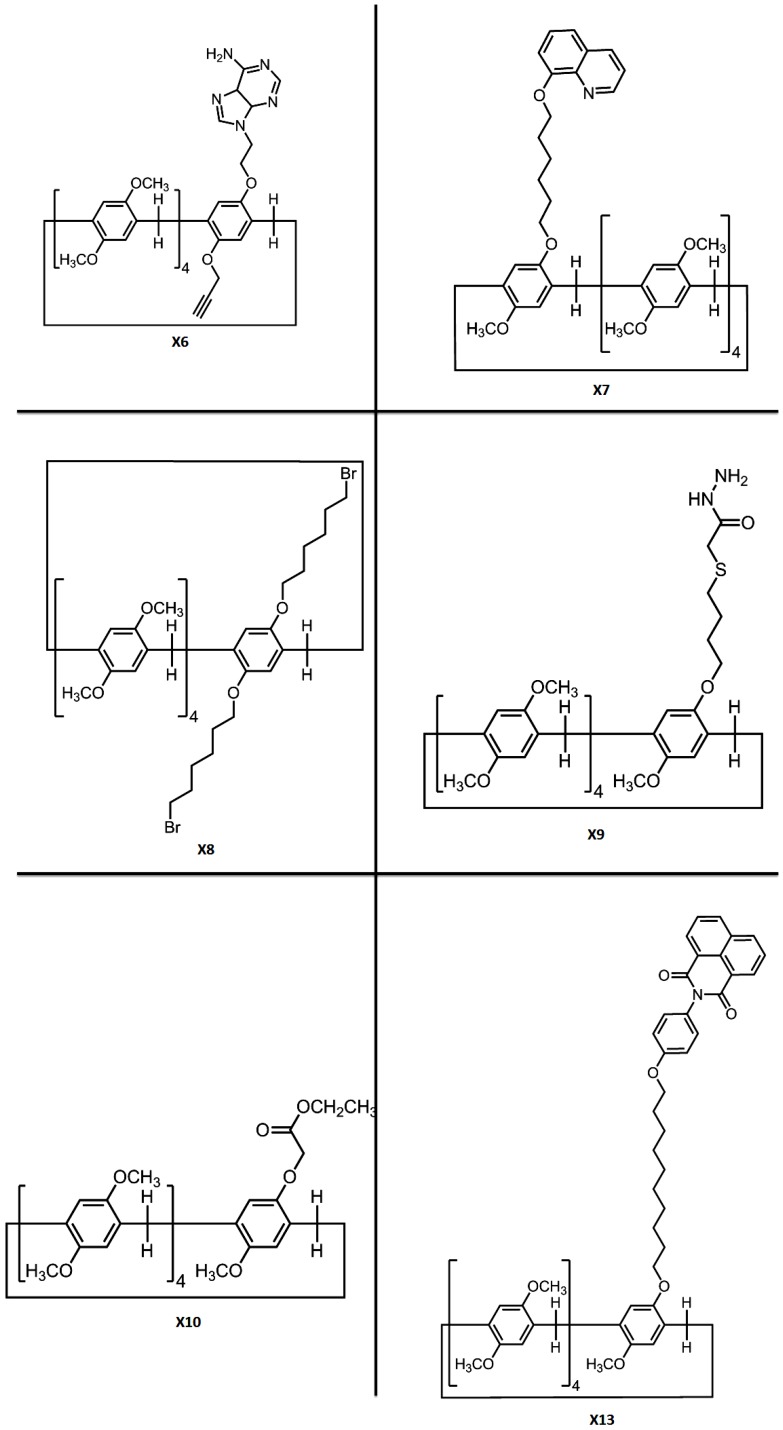
Chemical structures of precursors for the synthesis of pillararenes trimer, as well as other significant building blocks for the construction of assemblies (**X1**–**X13**).

In this review, we briefly summarized the recent progress about pillararenes trimers (**PT1–PT10** according to the timeline, Table 1 and Scheme 1), their synthesis method, supramolecular interactions with e.g., guest molecules (**G1–G9**, Scheme 2), driving forces for self-assembly, the fabrication of advanced self-assembly, and current applications. We will try to find the issues and concerns in the development of pillararenes trimers and their advanced self-assembly. For example, pillararenes trimer could be prepared via both covalent and noncovalent bonds. The selection of the synthesis strategy and functional precursors (Scheme 3 and Table 1) will become significant because it will further affect the molecular geometry including “star-shaped” molecules and linear foldamers, as well as driving forces for next-step self-assembly, e.g., by utilizing unoccupied pillararenes cavities via host-guest interactions or functional groups from modifications via other supramolecular interactions. In addition, the precise control over the self-assembly is always the key point during practical applications. Thus, we will analyze the sensitive supramolecular interactions of pillararenes trimers-based self-assemblies in details, e.g., which kind of supramolecular interactions will be involved in the construction process? Will they be sensitive towards external-stimuli such as variable concentrations, changed pH values, different temperature, as well as the addition/removal of competition guests and ions? Finally, we will also try to foresee future research directions in the field of pillararenes trimers-based self-assemblies.

## 2. Fabrication Strategy for Pillararenes Trimer 

Pillararenes trimer could be fabricated via both covalent and noncovalent bonds. To build the pillararenes trimer by covalent bonds, the classic organic synthesis reactions such as click reaction [29], palladium-catalyzed coupling reaction [4], and aminolysis reaction are employed. Additionally, the selection of “bridge” reagent is very important, for example, 1,3,5-benzenetricarbonyl trichloride is proved to be a very efficient “core” in the construction of star-shaped trimers by adopting various reactions such as amidation and esterification. Furthermore, the noncovalent method for preparing pillararenes trimers is mainly dependent on the supramolecular interactions such as host-guest interactions [9]. The roles of pillararenes in those noncovalent pillararenes trimers include performing as a linker in the self-assembled architecture and acting as a “wheel” subunit in mechanically interlocked molecules.

### 2.1. Synthesis by Organic Reactions 

“Click” reaction is a very efficient synthesis strategy for obtaining the “star-shaped” pillararenes trimer. For example, the first pillararenes trimer **PT1** (Scheme 1) was synthesized by using the general condition of click reactions, i.e., copper (I) catalyzed Huisgen-type azide-alkyne cycloaddition reaction between azido-pillar[5]arene (**X1**, Scheme 3) and tripropargylamine with a high yield of 91% [15]. By using the same synthesis strategy, **PT5** (Scheme 1) [22] is also produced by coupling the pillar[5]arenes precursors **X6** (Scheme 3) together.

Palladium-catalyzed coupling reactions such as Sonogashira reactions are also used for the fabrication of “rigid arms” of star-shaped trimers, for example, the general catalysis reagents i.e., CuI and dichlorobis(triphenylphosphine)palladium(II) were used in coupling alkynes together for the synthesis of **PT2** (Scheme 1) [20].

Amidation is another direct and simple method to afford pillararenes trimers. For example, **PT3** (Scheme 1) was synthesized by rationally connecting three units of pillararenes (**X4**, Scheme 3) together via the reaction between 1,3,5-benzenetricarbonyl trichloride and dihydrazide moieties in dichloromethane for 12 h [21]. By using very similar synthesis procedure but different pillar[5]arenes precursors such as **X9** (Scheme 3), 1,3,5-benzenetricarbonyl trichloride can also act as the core structure in another trimer—**PT7** (Scheme 1) [24]. In addition, the 1,3,5-benzenetricarbonyl trichloride could perform an esterification in chloroform with three equivalent of pillararenes precursor **X5** (Scheme 3) to obtain another star-shaped trimer, **PT4** (Scheme 1) [19]. Similarly, aminolysis of mono-ester derivative **X10** (Scheme 3) with *tris*(2-aminoethyl)amine in the toluene/methanol mixture could produce **PT8** (Scheme 1) [25].

Different from the above star-shaped pillararenes trimers, there is a particular linear pillararenes trimer, **PT6** (Scheme 1), which is synthesized by a quinoline monofunctionalized pillar [5] arene **X7** and bis-bromhexine functionalized **X8** (Scheme 3) in *n*-butanol [23]. Due to the possession of quinoline and pillararenes subunits, this “*N*-type” tri-pillararenes-based foldamer could contribute π–π stacking interaction sites for further self-assembly.

### 2.2. Preparation by Noncovalent Method

#### 2.2.1. Supramolecular Interactions

**Figure 1:**
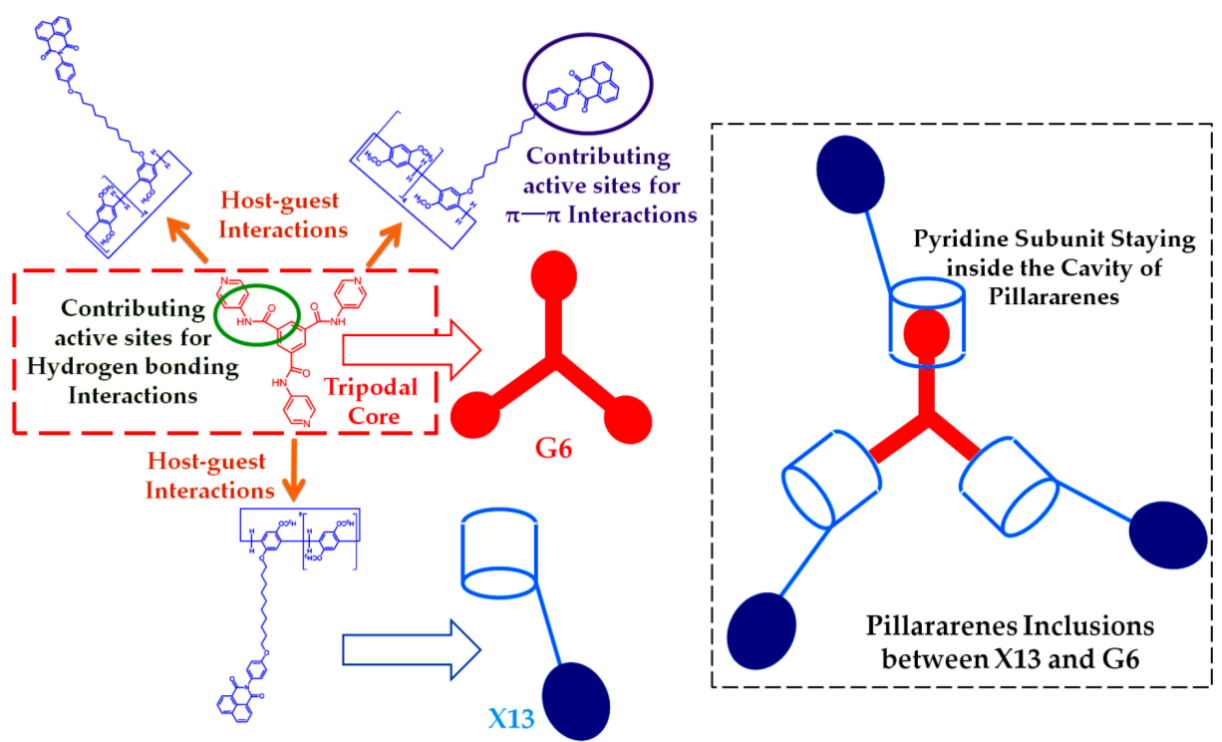
Proposed assembly mechanism of supramolecular inclusions **X13**⸧**G6** by noncovalent bonds [28].

A particular pillararenes trimer **X13**⸧**G6** (Figure 1) was designed by only employing noncovalent bonds between the cavity of naphthalimide monofunctionalized pillar [5] arene **X13** (Scheme 1) and pyridine moieties on the star-shaped guest **G6** (Scheme 2) [28]. In the noncovalent pillararenes trimer, the pillararenes subunit only acts as a linker to interact with the tripodal core, providing an occupied cavity for further control over the advanced self-assemblies.

#### 2.2.2. Mechanically Interlocked Molecules

**Figure 2:**
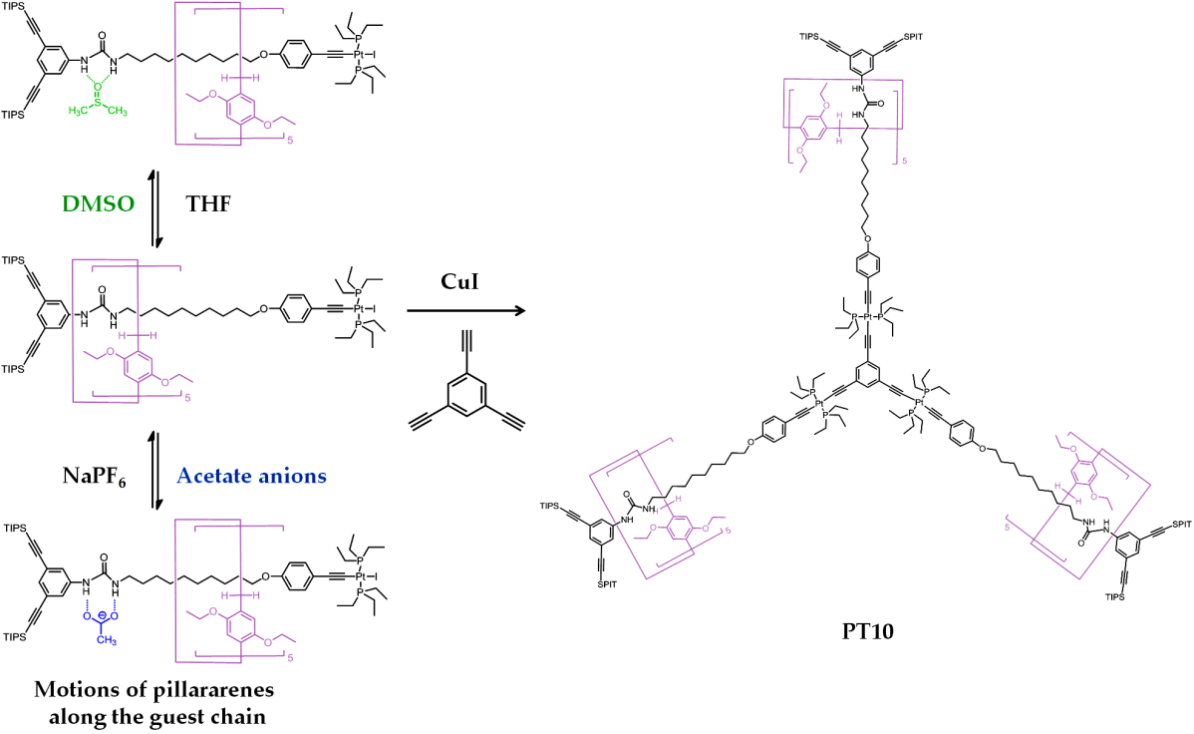
Synthesis of mechanically interlocked molecule **PT10** by the CuI-catalyzed coupling reaction between star-shaped molecule—1,3,5-triethynylbenzene and pseudorotaxane **X12**⸧**G9**, as well as the solvent/anion-induced switchable motions of the wheel—**X12** along neutral alkyl chain guest **G9** [27]. External stimuli responsiveness could be indicated by the chemical structural changes.

Mechanically interlocked molecules are fabricated by noncovalent bonds, but only can be destroyed by damaging the covalent bonds [3]. The design strategy of mechanically interlocked molecules is also employed to prepare the pillararenes trimers. For example, 1,4-dipropoxypillar[5]arenes **X11** (Scheme 3) performs as the wheel including neutral alkyl chain guest **G8** (Scheme 2) to form the pseudorotaxane, and then coupled with 1,3,5-triethynylbenzene into the first-generation rotaxane dendrimer **PT9** (Scheme 1) via platinum–acetylide bonds in a yield of 79% [26,30,31]. The role of pillar[5]arenes is very significant in the fabrication of mechanical dendrimers, enhancing the rigidity of the resultant rotaxane and reducing the possibility of self-folding. In a similar example, 1,4-diethoxypillar[5]arenes **X12** (Scheme 3) and another neutral guest **G9** (Scheme 2) also formed the pseudorotaxane via C–H^…^π interactions, which could further couple with 1,3,5-triethynylbenzene into dendrimers **PT10** (Scheme 1) in a yield of 92% (Figure 2) [27]. The chemical structure of **PT10** was confirmed by multinuclear (^1^H, ^31^P and ^13^C) NMR measurements, ESI-MS and gel permeation chromatography (GPC) spectra.

## 3. Pillararenes Trimer as Building Block for Fabricating External-Stimuli Responsive Self-Assembled Materials

Both covalent and noncovalent pillararenes trimers could act as the building blocks to construct advanced self-assembled materials. The driving forces for further self-assembly include host-guest interactions, charge transfer, hydrophobic, hydrogen bonding, and C–H^…^π and π–π stacking interactions. Due to the particular “star-shaped” geometry of pillararenes trimers, thus formed complicated self-assemblies are main supramolecular networks and frameworks. Interestingly, because those supramolecular materials are constructed by functionalized building blocks via supramolecular interactions, they could be controlled over morphologies, as well as formation/deformation by employing various external-stimuli such as variable concentrations, changed pH values, different temperature, as well as the addition/removal of competition guests and ions.

### 3.1. Interactions and Driving Forces

If host and guest molecules were both employed, host-guest interactions driven by charge transfer, hydrophobic and hydrogen bonding interactions become very general driving forces to prepare building blocks for the construction of complicated self-assemblies. For example, host-guest interaction between electronic deficient viologen moieties and the electronic rich cavity of pillararenes are employed as one significant driving force for the construction of self-assembled materials. A clear color change from colorless to yellow brown could be observed in the solution containing **PT1** upon the addition of **G1** (Scheme 2), due to the charge transfer in the formation of inclusions **PT1**⸧**G1** [15]. Similar phenomena could also be observed in the mixed solutions containing dimethoxypillar (**X2**, Scheme 3) and **G1** [15].

In another example, benzene-1,3,5-trispillar[5]arene **PT4** (Scheme 1) could effectively include other electron acceptors such as 1,4-butane diamine (**G3**, Scheme 2) and *tris*(2-aminoethyl)amine (**G4**, Scheme 2) via host-guest interactions [19]. Interestingly, driven by charge transfer interactions, **PT4**⸧**G3** and **PT4**⸧**G3** further exhibit clear aggregation-induced absorption in the visible to near IR regions (400–1000 nm), quite different from the behaviors shown by inclusions between pillararenes monomers/dimers and guests.

Additionally, the neutral guest moiety such as cyano and triazole group on **G2** (Scheme 2) [20] can be included by the cavity of pillararenes on **PT2**, and provide proper host-guest interactions to serve as the candidate of building blocks for making complicated self-assembly.

Except for using the cavity of pillararenes, the bridging moiety among those pillararenes units could also interact with guest molecules, for example, hydrogen bonding interactions [22] were found between the *tris*(2-aminoethyl)amine subunit on **PT8** and *N*-phenyl-3-(phenylimino)-3H-phenothiazin-7-amine (**G7**, Scheme 2) [25] as confirmed by ^1^H NMR.

The host-guest inclusion can further be confirmed by UV-vis spectra [19], ^1^H NMR titration [15,22], variable temperature ^1^H NMR [15,19], ^1^H-^1^H COSY [20], and ^1^H-^1^H NOSY [15,20], as well as high resolution time-of-flight mass spectrometry [15]. To better study host-guest interactions, several key parameters were involved, for example, the stoichiometry between host and guest molecules exhibit the composition ratio, and can be determined by Job’s variation method by fluorescence, UV-vis spectra, and cyclic voltammogram (CV) [15,19,22,25]. In addition, the association constant (*K_a_*) indicates the binding strength of inclusions, and is able to be detected by e.g., the Benesi–Hildebrand equation [15] and variable spectrometric methods [15,22,25].

If guest molecules were not employed in the process of self-assembly, pillararenes trimers could adopt other significant interactions as driving forces for building assemblies. For example, due to the possession of amide and benzene moieties, neighboring **PT3** (Scheme 1) molecules could interact with each other via diverse interactions in cyclohexanol solutions, such as intermolecular hydrogen bonding between –N–H and O=C– as confirmed by IR spectra, as well as C–H^…^π and π–π stacking interactions among pillararenes and phenyl units as indicated by X-ray powder diffusion (PXRD), ^1^H NMR, and IR spectra [21].

### 3.2. Multi-Dimensional Self-Assembly and Its External-Stimuli Responsiveness

Compared to pillararenes monomer, pillararenes trimer provides a larger possibility for the construction of multi-dimensional complicated self-assemblies with external stimuli responsiveness.

**Figure 3:**
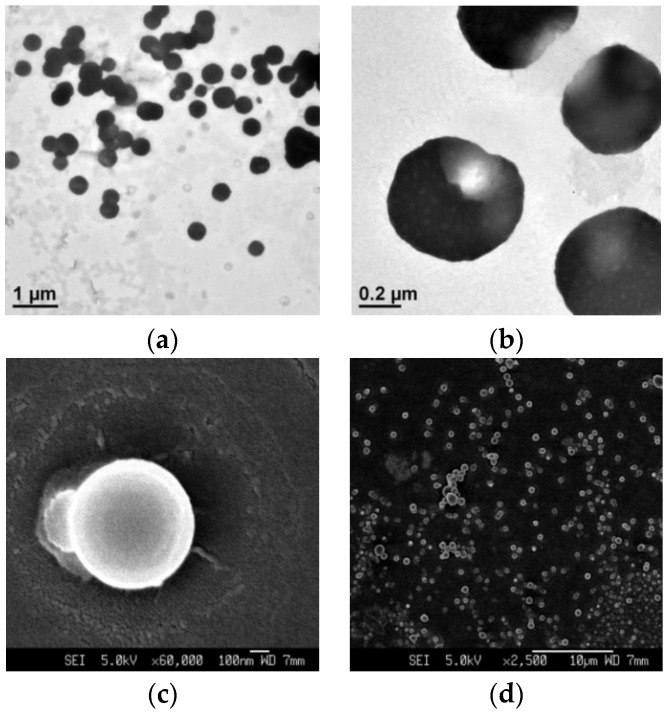
Negative-stained TEM images (**a**) scale bar = 1 μm and (**b**) scale bar = 0.2 μm and gold sputtering SEM images (**c**) scale bar = 10 μm and (**d**) scale bar = 100 nm about the vesicular assemblies by the inclusion **X2**⸧**G1** (1 × 10^−3^ mol L^−1^). Reproduced with permission from [15]. Copyright Royal Society of Chemistry, 2013.

**Figure 4:**
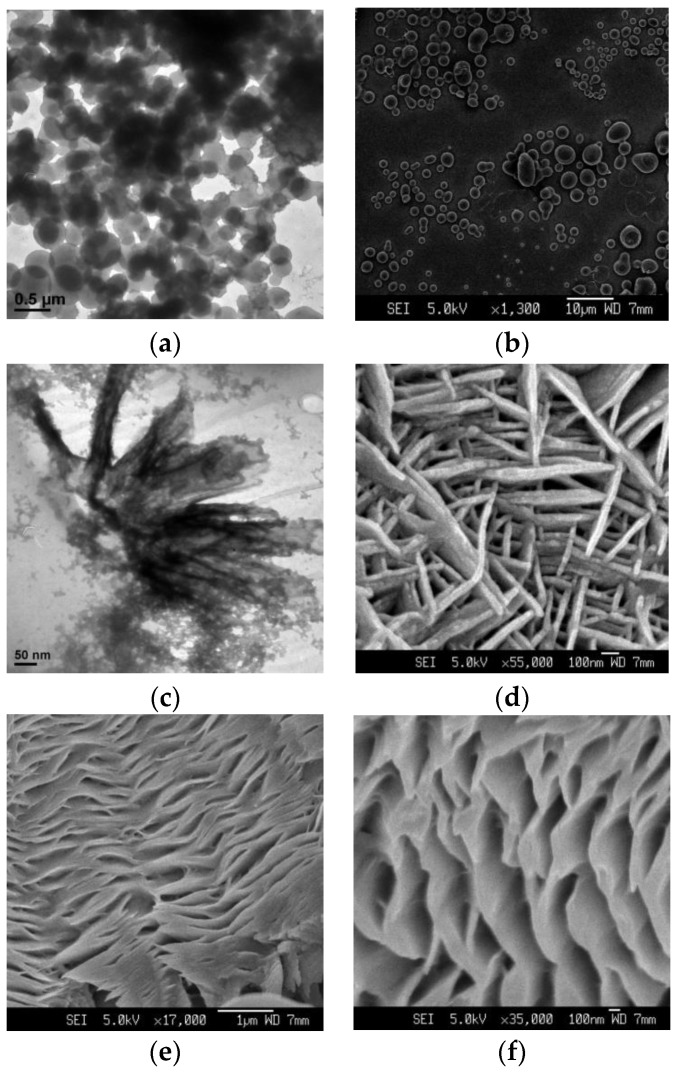
Concentration-dependent morphological control by the inclusion **PT1**⸧**G1**. (**a**) TEM (scale bar = 0.5 μm) and (**b**) SEM images (scale bar = 10 μm) under the sample concentration of 1 × 10^−3^ mol L^−1^. (**c**) TEM (scale bar = 50 nm) and (**d**) SEM images (scale bar = 100 nm) under the sample concentration of 2 × 10^−3^ mol L^−1^. (**e**,**f**) SEM images (scale bar = 1 μm and 100 nm) under the sample concentration of 5 × 10^−3^ mol L^−1^. Reproduced with permission from [15]. Copyright Royal Society of Chemistry, 2013.

For example, the pillararenes monomer—1,4-dimethoxypillar[5]arene (**X2**, Scheme 3) could include the guest molecule—biviologen (**G1**, Scheme 2) into a self-assembled amphiphile with solvophilic/solvophobic moieties, and further aggregate into spherical architectures, which show very limited morphological changes upon increasing concentrations as observed by transmission electron microscopy (TEM) and scanning electronic microscopy (SEM, Figure 3) [15]. The critical assembly concentrations (CAC) generated by UV-vis and fluorescence emitted spectra were also employed to exhibit the formation and possible changes of aggregations [15]. However, the star-shaped pillararenes trimer **PT1** (Scheme 1) could form stable supramolecular networks by complexing with **G1**, due to host-guest interactions. Upon the increasement of sample concentrations, those small pieces of supramolecular networks become larger and further wrap into spherical vesicles (zero dimensional, 0 D) as confirmed by TEM, SEM, and dynamic light scattering (DLS). Interestingly, those inclusions have the capacity of carrying out a morphological transformation from fused vesicular assembled structures, tubular objects (one dimensional, 1 D), layers (two dimensional, 2 D) to stacked layers (three dimensional, 3 D) upon continuously increasing the sample concentrations (Figure 4). Thus, the inclusions between **PT1** and **G1** exhibit an efficient concentration-dependent control over morphologies of assembled materials [15].

**Figure 5:**
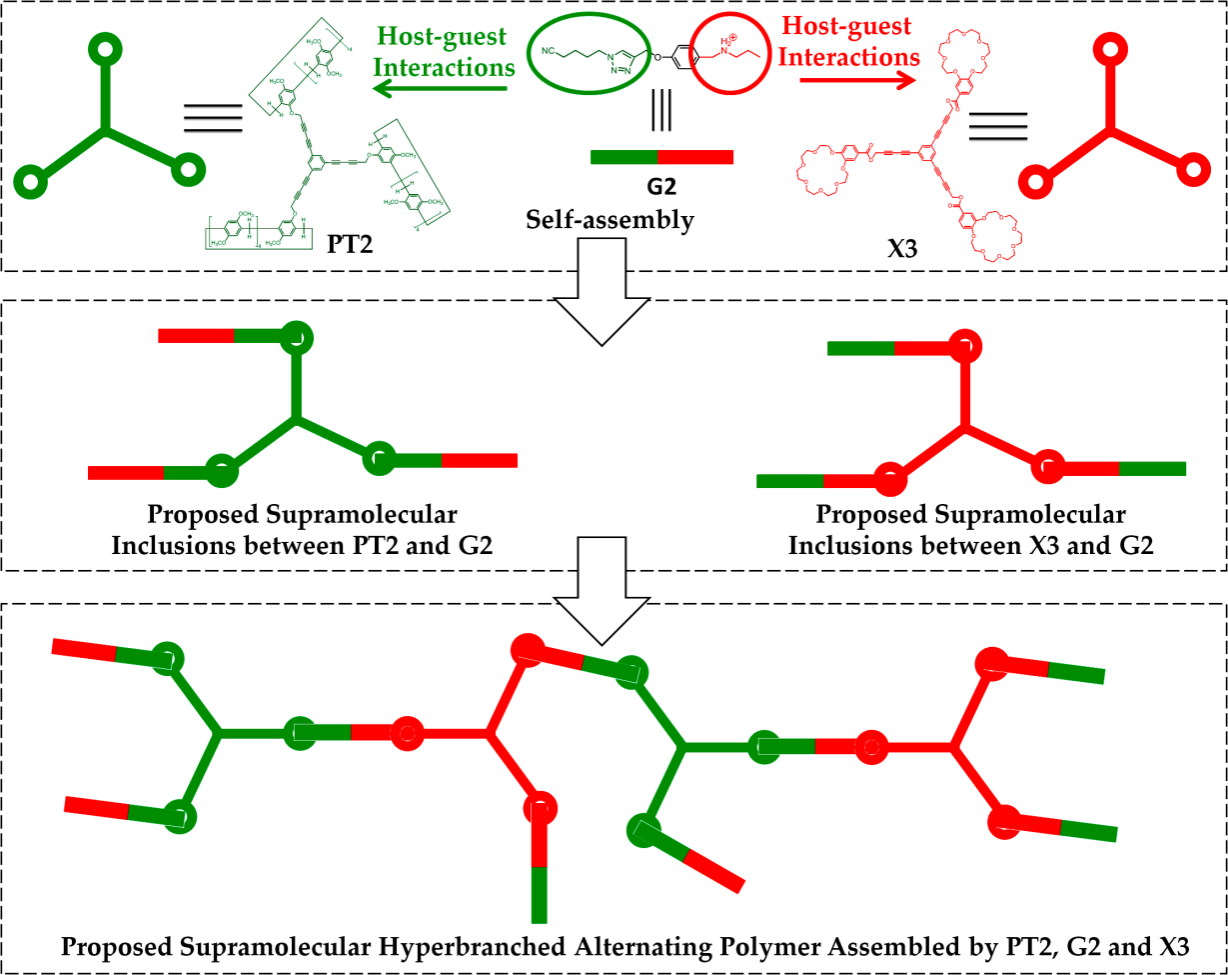
Graphical representation of the formation of supramolecular hyperbranched alternating polymer [20].

Except for tuning samples concentration of building blocks such as PT1⸧G1 [15], the addition and removal of cations were also proved to be an efficient method for controlling the formation/deformation of self-assemblies. For example, the pillararenes trimer PT2 (Scheme 1) can selectively bind the neutral moiety, cyano, and triazole groups on the guest molecule—G2 (Scheme 2), while the dialkylammonium groups on G2 are recognized by the cavities on crown ether trimers (X3, Scheme 3). The formation of supramolecular hyperbranched alternating polymers by PT2, G2, and X3 in a molar ration of 1/3/1 (Figure 5) was proved by diffusion-ordered NMR spectroscopy (DOSY), DLS, and TEM. The critical polymerization concentration (CPC) was calculated as 6 × 10^−3^ mol L^−1^ by a double logarithmic plot of specific viscosity versus the concentration of G2. Due to the competition of metal cations such as K^+^ [20], the inclusion and exclusion of dialkylammonium groups on G2 by the crown ether moieties on X3 can be used as a tool to control the assembly and disassembly of those supramolecular polymeric materials.

**Figure 6:**
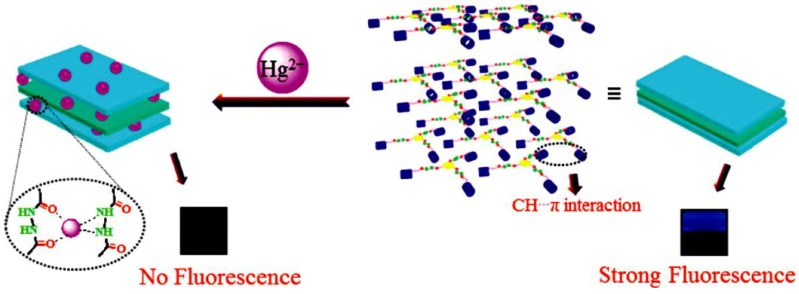
Graphic representation of **PT3**-based self-assemblies and their responsiveness towards Hg^2+^. Reproduced with permission from [21]. Copyright Royal Society of Chemistry, 2018.

In another example, supramolecular polymers prepared by **PT3** (Scheme 1) in cyclohexanol solution via hydrogen bonding, van der Waals forces, and C–H^…^π and π–π stacking interactions can also be responsive to metal cations [21]. It is found that **PT3**-based supramolecular materials possess the lowest critical gelation concentration (CGC) as 5% (w/v, 10 mg mL^−1^ = 1%) and the gel-sol transition temperature (*T_gel_*) as 58–60 °C, as well as exhibit an aggregation-induced emission (AIE). Due to the coordination between dihydrazide moieties on **PT3** and metal cations such as Hg^2+^, the intermolecular hydrogen bonds are affected, leading to quench of AIE and the disassociation of supramolecular polymers (Figure 6). Furthermore, the AIE supramolecular organic framework gel prepared by thioacetylhydrazine-bearing pillar[5]arenes trimer **PT7** (Scheme 1) and 4-aminopyridine-functionalized trimeric amide **G6** (Scheme 2) via hydrogen bonding, C–H^…^π and π–π stacking interactions could exhibit fluorescent response for multiple metal cations such as Fe^3+^, Cu^2+^, Cr^3+^, Ag^+^, Tb^3+^, and Eu^3+^ [24].

Except for response to metal cations, pillararenes trimer-based material can also be responsive to anion and solvent molecules. For example, due to C–H^…^π interactions between pillararenes and alkyl chain in **PT10** (Scheme 1), the urea subunit localizes inside the cavity of pillararenes [27]. Upon the addition of hydrogen bonding acceptors such as dimethylsulfoxide and acetate anion, the controllable motion of the pillararenes wheel to methylene subunit is achieved, finally leading to the dimension modulation of dendrimers (Figure 2).

In addition, pillararenes trimers-based self-assembly could be responsive to other external stimuli such as pH and temperature changes. For example, the pillar[5]arenes trimer bearing adenine subunits (**PT5**, Scheme 1) could include the uracil derivative—6-(2,4-dioxo-3,4-dihydropyrimidin-1(2*H*)-yl)hexanenitrile (**G5**, Scheme 2) via host-guest interactions, and the neighboring **G5** could further have hydrogen bonding and π–π stacking interactions with adenine subunits on **PT5**, leading to the formation of hyperbranched supramolecular polymers [22]. Due to the formation and deformation of hydrogen bonding interactions between uracil and adenine subunits, the assembly and disassembly of supramolecular polymers could be controlled by the addition of acids such as aspirin and bases such as triethylamine, respectively. Furthermore, because host-guest and hydrogen bonding interactions are sensitive to temperature changes, the supramolecular polymers **PT5**⸧**G5** could be affected by heating and cooling.

## 4. Applications

Pillararenes trimers-based supramolecular self-assemblies provide a possibility in the construction of multi-dimensional materials [15], exhibit interesting external stimuli responsiveness, and could further be used for diverse applications such as detection, sorption, and separation of significant multi-analytes including metal cations, anions, and amino acids.

**Figure 7:**
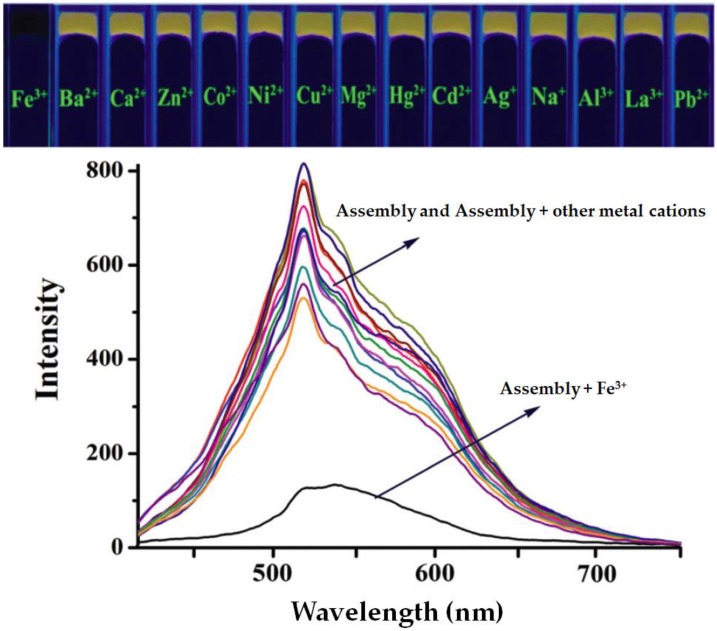
Fluorescence spectra of **X13**⸧**G6** with the addition of various metal cations in aqueous solutions. Reproduced with permission from [28]. Copyright Royal Society of Chemistry, 2019.

For example, the **PT3**-based supramolecular materials with AIE properties exhibit selective response towards Hg^2+^ with the detection limit (LOD) of 1.02 × 10^−8^ mol L^−1^ as calculated by fluorescence titration, which can be used as an ultrasensitive sensor. Furthermore, the separation rate for Hg^2+^ was proved to be 81.3% by inductively coupled plasma (ICP) [21], confirmed the ingestion capacity of those supramolecular materials for particular metal cations. Similar application for detecting metal cations can also be found by other supramolecular self-assembled materials such as **PT6**-based supramolecular networks [23], i.e., by the assistance of π–π stacking interactions, the linear pillararenes trimer-based foldamer **PT6** (Scheme 1) could assemble into AIE supramolecular organic frameworks (SOFs) with ultrasensitive response for Fe^3+^/Hg^2+^/Cr^3+^ with the limits of detection in the range of 9.40 × 10^−10^–1.86 × 10^−9^ mol L^−1^ [23]. The AIE self-assemblies based on noncovalent pillararenes trimer—**X13**⸧**G6** via hydrogen bonding, π–π stacking and host-guest interactions (Figure 1 and Figure 7) could also ultra-sensitively detect Fe^3+^ with the limits of detection as 9.0 × 10^−10^ mol L^−1^, and exhibit separation properties towards Fe^3+^ [28] with a separation rate up to 99.8%.

Except for detecting metal cations, the host-guest inclusions between pillararenes trimer **PT8** and *N*-phenyl-3-(phenylimino)-3*H*-phenothiazin-7-amine (**G7**) could perform as a colorimetric probe for F^−^/AcO^−^/H_2_PO_4_^−^, due to the competition binding of anions with proton donating amide groups on **PT8** in comparison with **G7** [25]. Furthermore, the self-assembled materials could perform better ultrasensitive detection towards anions. For example, the **PT6**-based AIE supramolecular organic frameworks could further coordinate with metal cations such as Fe^3+^/Hg^2+^/Cr^3+^, leading to the formation of metal ions coordinated supramolecular organic frameworks such as **PT6**⸧Fe^3+^, **PT6**⸧Hg^2+^, and **PT6**⸧Cr^3+^. Due to the possession of stronger binding constants between metal cations and specialized anions, those metal ligated materials could further carry out selective fluorescence “turn-on” ultrasensitive detection towards anions such as CN^−^ and H_2_PO_4_^−^, giving the limits of detection as 2.12 × 10^−9^ and 1.78 × 10^−9^ mol L^−1^, respectively [23].

Due to the capacity of multiple responsiveness to metal cations, anions, and amino acid, the **PT7**-based AIE supramolecular organic framework gels and metallogels could behave as a multi-unit sensor array for Fe^3+^, Cu^2+^, Cr^3+^, Ag^+^, Tb^3+^, Eu^3+^, F^−^, CN^−^, HSO_4_^−^, histidine (His), serine (Ser), and cysteine (Cys) [24] with the limits of detection ranging from 1.20 × 10^−8^ to 6.80 × 10^−10^ mol L^−1^. Particularly, the detection for Fe^3+^, Cr^3+^, Tb^3+^, Eu^3+^, F^−^, CN^−^, and serine could achieve an ultrasensitive level. Furthermore, the adsorption percentages of those supramolecular materials for Fe^3+^, Cu^2+^, Cr^3+^, Ag^+^, Tb^3+^, and Eu^3+^ could reach 98.30%, 99.34%, 98.80%, 99.57%, 98.30%, and 98.40%, respectively.

Those AIE supramolecular assemblies shown above such as polymeric gels not only provide an interesting strategy for building external stimuli responsible materials, but also are expected to make contributions to molecular sensors, biological imaging and sensing, as well as controlled drug delivery.

## 5. Overview and Outlook

Pillararenes trimer has been proved to an efficient building blocks in the construction of supramolecular self-assembled materials. Pillararenes trimers could be “star-shaped” molecules and linear foldamers, which are fabricated via covalent bonds by employing classic organic synthesis reactions such as “click”, palladium-catalyzed coupling, amidation, esterification, and aminolysis reaction. Interestingly, the “core” reagent—1,3,5-benzenetricarbonyl trichloride is very popular as the bridge to covalently connect three equivalents of pillararenes subunits together in the construction of “star-shaped” trimers. Alternatively, due to the capacity of recognizing guest molecules via host-guest interactions, pillararenes could perform as the linker and the “wheel” subunit in the construction of self-assembled pillararenes trimers and mechanically interlocked molecules, respectively. Thus, obtained pillararenes trimers could further act as building blocks for advanced self-assembled materials including supramolecular networks and frameworks via host-guest interactions, charge transfer, hydrophobic, hydrogen bonding, and C–H^…^π and π–π stacking interactions. Particularly, because those supramolecular materials are constructed by functionalized building blocks and sensitive supramolecular interactions, their morphologies and formation/deformation could be controlled by employing various external-stimuli such as variable concentrations, changed pH values, different temperature, as well as the addition/removal of competition guests and ions. Due to the external stimuli-responsiveness, pillararenes trimers-based supramolecular self-assemblies also become a significant candidate for diverse applications such as detection, sorption, and separation of significant multi-analytes including metal cations, anions, and amino acids.

A lot of perspective work in this area is still attractive for researchers in synthesis and material sciences, for example, (1) the design of new candidates for pillararenes trimers can be improved by choosing larger-sized pillararenes as the precursors, e.g., pillar[6]arenes [12], which also has good synthesis yields. Currently, only pillar[5]arenes is employed in the fabrication of pillararenes trimers. (2) the geometry of pillararenes trimers should be diverse, e.g., linear foldamer. Up to now, there was only one example about adopting linear pillararenes trimer for self-assembly [23]. (3) the study of mechanism about self-assemblies is unsatisfied. More researches should be employed, such as theoretical studies. The significant intermediates during self-assembly should be captured and better proved by using e.g., X-ray crystallography analysis. (4) alternative building blocks integrated with multi-subunits of pillararenes such as pillararenes tetramers [32,33,34,35,36,37] and other significant pillararenes oligomers [38,39] should be designed based on the researches of pillararenes trimers, as well as applied for recognizing multiple guests and fabricating assembled materials. (5) except for synthesizing novel pillararenes trimers, preparing trimeric guest molecules [40,41,42,43] is another alternative method to build planar assembled networks [19,24]. It is expected that future researches in the field of pillararenes trimers-based supramolecular assemblies will achieve to a new level in not only fundamental studies but also in practical applications in our lives.

## Figures and Tables

**Table 1:** Comparison of various pillararenes trimer (**PT1**–**PT10**), guest molecules (**G1**–**G9**), precursors, and other significant building blocks (**X1**–**X13**) for self-assembly and external stimuli responsiveness.

Pillararenes Trimer	Guest	Precursor	Interactions	Assembly	External Stimuli	Applications	Ref
PT1	G1	X1	Host-guest interactions	Hollow spherical, tubular and layered assemblies	Concentration-dependent	Morphological control in comparison with **X2**	[15]
PT2	G2	-	Host-guest interactions	Supramolecular hyperbranched alternating polymers	K^+^ (crown ether **X3**)	-	[20]
PT3	-	X4	Hydrogen bonding, van der Waals forces, C–H^…^π and π–π stacking interactions	Supramolecular polymer	Cations	Fluorescence detection and separation of Hg^2+^	[21]
PT4	G3/G4	X5	Host-guest interactions	Supramolecular polymer	-	-	[19]
PT5	G5	X6	Hydrogen bonding, π–π stacking and host-guest interactions	Hyperbranched supramolecular polymer	Heat and acid/base	-	[22]
PT6	-	X7/X8	π–π stacking interactions	(Metal ions coordinated) supramolecular organic frameworks	Fe^3+^/Hg^2+^/Cr^3+^ and CN^−^/H_2_PO_4_^−^	Fluorescence ultrasensitive detection	[23]
PT7	G6	X9	Hydrogen bonding, C–H^…^π and π–π stacking interactions	Supramolecular polymer network/supramolecular polymer framework	Metal cations/anions/amino acid	Fluorescence detection/adsorption capacity for cations	[24]
PT8	G7	X10	-	-	Competitive complexation with Anions	Fluorescence detection of F^−^/AcO^−^/H_2_PO_4_^−^	[25]
PT9	G8	X11	Mechanical interlocked molecule	Dendrimer	-	-	[26]
PT10	G9	X12	Mechanical interlocked molecule	Dendrimer	Dimethylsulfoxide and acetate anion	-	[27]
X13⸧G6	G6	X13	Hydrogen bonding, π–π stacking and host-guest interactions	Supramolecular polymer networks/gel	Heat/cooling, pH, competitive guests and mechanical	Dye sorption, ultrasensitive detection and separation of Fe^3+^	[28]

## Abbreviations

0 D	Zero dimensional
1 D	One dimensional
2 D	Two dimensional
3 D	Three dimensional
CAC	Critical assembly concentrations
CGC	Critical gelation concentration
CPC	Critical polymerization concentration
CV	Cyclic voltammogram
Cys	Cysteine
DLS	Dynamic light scattering
DOSY	Diffusion-ordered NMR spectroscopy
GPC	Gel permeation chromatography
His	Histidine
ICP	Inductively coupled plasma
*K_a_*	Association constant
LOD	The detection limit
PXRD	X-ray powder diffusion
SEM	Scanning electronic microscopy
Ser	Serine
SOFs	Supramolecular organic frameworks
TEM	Transmission electron microscopy
*T_gel_*	Gel-sol transition temperature

## References

[1] Yu G., Jie K., Huang F. (2015). Supramolecular Amphiphiles Based on Host-Guest Molecular Recognition Motifs. Chem. Rev..

[2] Sun Y., Chen C., Stang P.J. (2019). Soft Materials with Diverse Suprastructures via the Self-Assembly of Metal-Organic Complexes. Acc. Chem. Res..

[3] Nierengarten I., Deschenaux R., Nierengarten J.F. (2016). From Pillar[n]arene Scaffolds for the Preparation of Nanomaterials to Pillar[5]arene-containing Rotaxanes. Chimia.

[4] Zhang H., Lee J., Lammer A.D., Chi X., Brewster J.T., Lynch V.M., Li H., Zhang Z., Sessler J.L. (2016). Self-Assembled Pyridine-Dipyrrolate Cages. J. Am. Chem. Soc..

[5] Zhang H., Lee J., Brewster J.T., Chi X., Lynch V.M., Sessler J.L. (2019). Cation-based Structural Tuning of Pyridine Dipyrrolate Cages and Morphological Control over Their Self-assembly. J. Am. Chem. Soc..

[6] Murray J., Kim K., Ogoshi T., Yao W., Gibb B.C. (2017). The aqueous supramolecular chemistry of cucurbit[n]urils, pillar[n]arenes and deep-cavity cavitands. Chem. Soc. Rev..

[7] Zhang Y.-M., Xu Q.-Y., Liu Y. (2019). Molecular recognition and biological application of modified β-cyclodextrins. Sci. Chin. Chem..

[8] Zhang H., Zou R., Zhao Y. (2015). Macrocycle-based metal-organic frameworks. Coordin. Chem. Rev..

[9] Wang Y., Ping G., Li C. (2016). Efficient complexation between pillar[5]arenes and neutral guests: From host-guest chemistry to functional materials. Chem. Commun..

[10] Guo F., Sun Y., Xi B., Diao G. (2018). Recent progress in the research on the host-guest chemistry of pillar[n]arenes. Supramol. Chem..

[11] Ogoshi T., Yamagishi T.-a., Nakamoto Y. (2016). Pillar-Shaped Macrocyclic Hosts Pillar[n]arenes: New Key Players for Supramolecular Chemistry. Chem. Rev..

[12] Cao D., Meier H. (2015). Synthesis of Pillar[6]arenes and Their Host–Guest Complexes. Synthesis.

[13] Xiao T., Wang L. (2018). Recent advances of functional gels controlled by pillar[n]arene-based host–guest interactions. Tetrahedron Lett..

[14] Ogoshi T., Kakuta T., Yamagishi T.-a. (2019). Applications of Pillar[n]arene-Based Supramolecular Assemblies. Angew. Chem. Int. Ed..

[15] Zhang H., Nguyen K.T., Ma X., Yan H., Guo J., Zhu L., Zhao Y. (2013). Host-guest complexation driven dynamic supramolecular self-assembly. Org. Biomol. Chem..

[16] Kakuta T., Yamagishi T.A., Ogoshi T. (2018). Stimuli-Responsive Supramolecular Assemblies Constructed from Pillar[n]arenes. Acc. Chem. Res..

[17] Zhang H., Liu Z., Zhao Y. (2018). Pillararene-based self-assembled amphiphiles. Chem. Soc. Rev..

[18] Xiao T., Zhong W., Xu L., Sun X.Q., Hu X.Y., Wang L. (2019). Supramolecular vesicles based on pillar[n]arenes: Design, construction, and applications. Org. Biomol. Chem..

[19] Liu L.-Z., Qin X., Duan W.-G., Huang H.-F., Zhang W.-X., Zhou Q.-Q., Huang Y. (2018). Aggregation-induced near-infrared absorption of a pillar[5]arene trimer by charge transfer interaction. Dyes Pigm..

[20] Li H., Fan X., Shang X., Qi M., Zhang H., Tian W. (2016). A triple-monomer methodology to construct controllable supramolecular hyperbranched alternating polymers. Polym. Chem..

[21] Jiang X.-M., Huang X.-J., Song S.-S., Ma X.-Q., Zhang Y.-M., Yao H., Wei T.-B., Lin Q. (2018). Tri-pillar[5]arene-based multi-stimuli-responsive supramolecular polymers for fluorescence detection and separation of Hg^2+^. Polym. Chem..

[22] Jiang Y.-Q., Wu K., Zhang Q., Li K.-Q., Li Y.-Y., Xin P.-Y., Zhang W.-W., Guo H.-M. (2018). A dual-responsive hyperbranched supramolecular polymer constructed by cooperative host–guest recognition and hydrogen-bond interactions. Chem. Commun..

[23] Wei T.-B., Ma X.-Q., Fan Y.-Q., Jiang X.-M., Dong H.-Q., Yang Q.-Y., Zhang Y.-F., Yao H., Lin Q., Zhang Y.-M. (2019). Aggregation-induced emission supramolecular organic framework (AIE SOF) gels constructed from tri-pillar[5]arene-based foldamer for ultrasensitive detection and separation of multi-analytes. Soft Matter.

[24] Liu J., Fan Y.-Q., Song S.-S., Gong G.-F., Wang J., Guan X.-W., Yao H., Zhang Y.-M., Wei T.-B., Lin Q. (2019). Aggregation-Induced Emission Supramolecular Organic Framework (AIE SOF) Gels Constructed from Supramolecular Polymer Networks Based on Tripodal Pillar[5]arene for Fluorescence Detection and Efficient Removal of Various Analytes. ACS Sustain. Chem. Eng..

[25] Khadieva A., Gorbachuk V., Shurpik D., Stoikov I. (2019). Synthesis of Tris-pillar[5]arene and Its Association with Phenothiazine Dye: Colorimetric Recognition of Anions. Molecules.

[26] Wang W., Chen L.J., Wang X.Q., Sun B., Li X., Zhang Y., Shi J., Yu Y., Zhang L., Liu M. (2015). Organometallic rotaxane dendrimers with fourth-generation mechanically interlocked branches. Proc. Natl. Acad. Sci. USA.

[27] Wang X.Q., Wang W., Li W.J., Chen L.J., Yao R., Yin G.Q., Wang Y.X., Zhang Y., Huang J., Tan H. (2018). Dual stimuli-responsive rotaxane-branched dendrimers with reversible dimension modulation. Nat. Commun..

[28] Guan X.-W., Lin Q., Zhang Y.-M., Wei T.-B., Wang J., Fan Y.-Q., Yao H. (2019). Pillar[5]arene-based spongy supramolecular polymer gel and its properties in multi-responsiveness, dye sorption, ultrasensitive detection and separation of Fe^3+^. Soft Matter.

[29] Kakuta T., Yamagishi T., Ogoshi T. (2017). Supramolecular chemistry of pillar[n]arenes functionalised by a copper(I)-catalysed alkyne-azide cycloaddition “click” reaction. Chem. Commun..

[30] Chen C. (2015). High-generation organometallic rotaxane dendrimer. Sci. Chin..

[31] Wang Y.-X., Zhou Q.-F., Chen L.-J., Xu L., Wang C.-H., Li X., Yang H.-B. (2018). Facile construction of organometallic rotaxane-terminated dendrimers using neutral platinum-acetylides as the main scaffold. Chem. Commun..

[32] Song N., Chen D.X., Qiu Y.C., Yang X.Y., Xu B., Tian W., Yang Y.W. (2014). Stimuli-responsive blue fluorescent supramolecular polymers based on a pillar[5]arene tetramer. Chem. Commun..

[33] Wu J., Sun S., Feng X., Shi J., Hu X.Y., Wang L. (2014). Controllable aggregation-induced emission based on a tetraphenylethylene-functionalized pillar[5]arene via host-guest recognition. Chem. Commun..

[34] Jin X.Y., Song N., Wang X., Wang C.Y., Wang Y., Yang Y.W. (2018). Monosulfonicpillar[5]arene: Synthesis, Characterization, and Complexation with Tetraphenylethene for Aggregation-Induced Emission. Sci. Rep..

[35] Liu Y., Shi B., Wang H., Shangguan L., Li Z., Zhang M., Huang F. (2018). Construction of Metallacage-Cored Supramolecular Gel by Hierarchical Self-Assembly of Metal Coordination and Pillar[5]arene-Based Host-Guest Recognition. Macromol. Rapid Commun..

[36] Song N., Lou X.Y., Hou W., Wang C.Y., Wang Y., Yang Y.W. (2018). Pillararene-Based Fluorescent Supramolecular Systems: The Key Role of Chain Length in Gelation. Macromol. Rapid Commun..

[37] Ali W., Ning G., Hassan M., Gong W. (2019). Construction of Pillar[5]arene Tetramer-Based Cross-Linked Supramolecular Polymers through Hierarchical Charge-Transfer and Host-Guest Interactions. Asian J. Org. Chem..

[38] Li Z.Y., Zhang Y., Zhang C.W., Chen L.J., Wang C., Tan H., Yu Y., Li X., Yang H.B. (2014). Cross-linked supramolecular polymer gels constructed from discrete multi-pillar[5]arene metallacycles and their multiple stimuli-responsive behavior. J. Am. Chem. Soc..

[39] Zhang C.-W., Ou B., Jiang S.-T., Yin G.-Q., Chen L.-J., Xu L., Li X., Yang H.-B. (2018). Cross-linked AIE supramolecular polymer gels with multiple stimuli-responsive behaviours constructed by hierarchical self-assembly. Polym. Chem..

[40] Li C., Han K., Li J., Zhang Y., Chen W., Yu Y., Jia X. (2013). Supramolecular Polymers Based on Efficient Pillar[5]arene-Neutral Guest Motifs. Eur. J. Chem..

[41] Li Q., Han K., Li J., Jia X., Li C. (2015). Synthesis of dendrimer-functionalized pillar[5]arenes and their self-assembly to dimeric and trimeric complexes. Tetrahedron Lett..

[42] Wu G.Y., Wang X.Q., Chen L.J., Hu Y.X., Yin G.Q., Xu L., Jiang B., Yang H.B. (2018). Supramolecular Polymer Cross-Linked by Discrete Tris-[2]pseudorotaxane Metallacycles and Its Redox-Responsive Behavior. Inorg. Chem..

[43] Zhang C.-W., Jiang S.-T., Yin G.-Q., Li X., Zhao X.-L., Yang H.-B. (2018). Dual Stimuli-Responsive Cross-Linked AIE Supramolecular Polymer Constructed through Hierarchical Self-Assembly. Isr. J. Chem..

